# Protostylid: Contributing and Aggravating Factor for Periodontitis

**DOI:** 10.7759/cureus.58347

**Published:** 2024-04-15

**Authors:** Sanket Shinde, Ramnath Elangovan, Dinesh C Maganti, Danilo Milanes Zambrano, Collins N Agholor, Anwesha Das

**Affiliations:** 1 Periodontology, Ram Krishna Dharmarth Foundation University, Bhopal, IND; 2 Periodontology, School of Dentistry, University of Rwanda, Kigali, RWA; 3 Pediatric Dentistry and Orthodontics, School of Dentistry, University of Rwanda, Kigali, RWA; 4 Prosthodontics and Restorative Dentistry, School of Dentistry, University of Rwanda, Kigali, RWA; 5 Periodontology, Sri Aurobindo College of Dentistry, Indore, IND

**Keywords:** dental forensics, endodontic therapy, protostylid, periodontitis, plaque accumulation

## Abstract

Clinicians should be well-versed in the anatomy, variations, and teeth anomalies. Developmental disturbances of the teeth can lead to alterations in size, shape, number, structure, and eruption of the teeth. Developmental disturbances can lead to germination, fusion, concrescence, dilaceration, talons, cusps, dens in dente, etc. Protostylid, an additional cusp on the buccal aspect of the maxillary molar, which is a rare clinical finding, can lead to plaque accumulation, making oral hygiene maintenance difficult. This leads to clinical attachment loss and bone loss. This condition may often go undiagnosed. It should be diagnosed to prevent further complications. This case has been reported to make clinicians aware of the importance of diagnosing the case at the earliest possible time so that complications can be prevented and management is easier. From the perspective of forensic dentistry, this morphological feature, though uncommon, may be useful for the classification and identification of victims in mass causalities and bite marks on bodies or inanimate objects. This is one of the rarest cases of protostylids reported to date. This may not only pose a significant problem in endodontic therapy due to morphological alterations in root canals and periodontal therapy due to grove formation leading to an inability to maintain a plaque-free area (bone loss) but also be of very significant interest from the perspective of forensic dentistry.

## Introduction

Identifying and characterizing dental anomalies such as protostylids and talon cusps is crucial for clinical and anthropological purposes. In clinical settings, thorough examination and diagnosis of such variations are essential to ensure proper treatment planning and management and prevent potential complications. For instance, the presence of a protostylid or talon cusp may affect occlusal relationships, predispose individuals to dental caries or periodontal disease, and impact overall oral health and function. Additionally, understanding the prevalence and distribution of these anomalies across different populations contributes to our knowledge of human variation and evolution. Anthropological studies investigating the frequency and distribution of protostylids and talon cusps among various ethnic groups provide insights into genetic and environmental factors influencing dental morphology and development [1]. By integrating clinical observations with anthropological research, we can gain a comprehensive understanding of the significance and implications of dental anomalies in contemporary and historical populations.

## Case presentation

**Figure 1:**
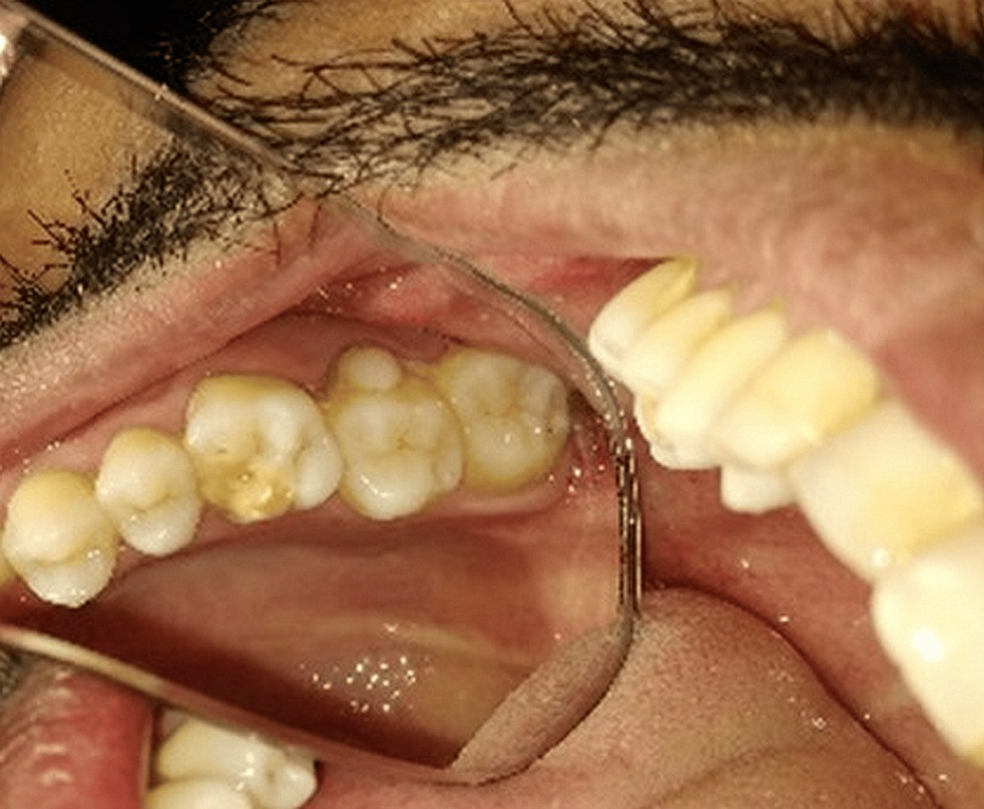
Tooth number 17 showing supernumerary cusp on the buccal aspect of the molar in occlusal view

**Figure 2:**
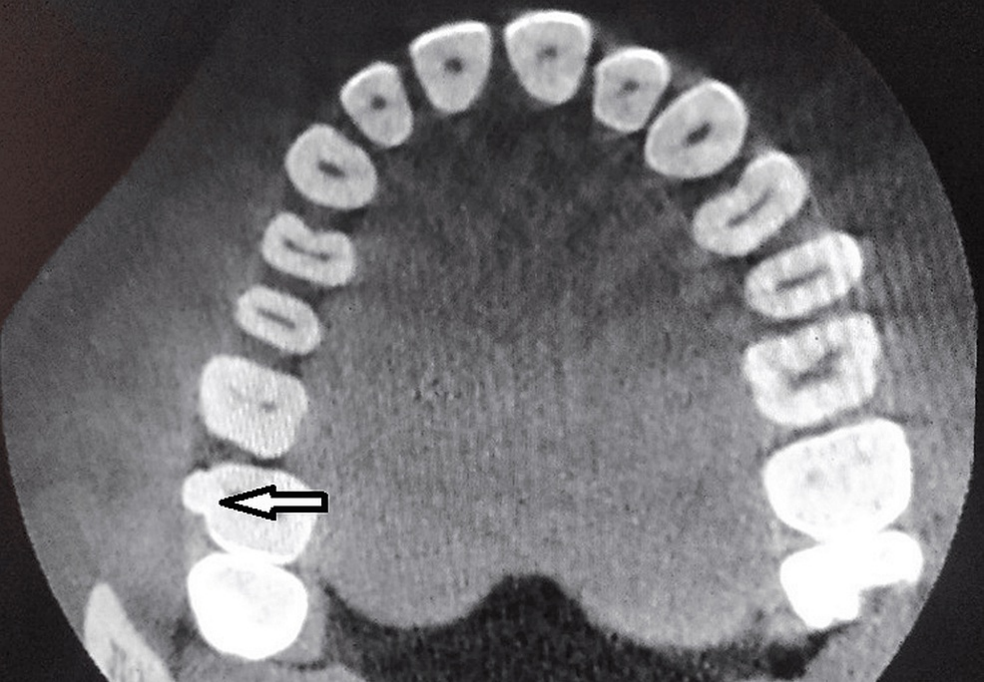
Axial view at the coronal aspect of tooth number 17 showing supernumerary cusp and pulp horns

**Figure 3:**
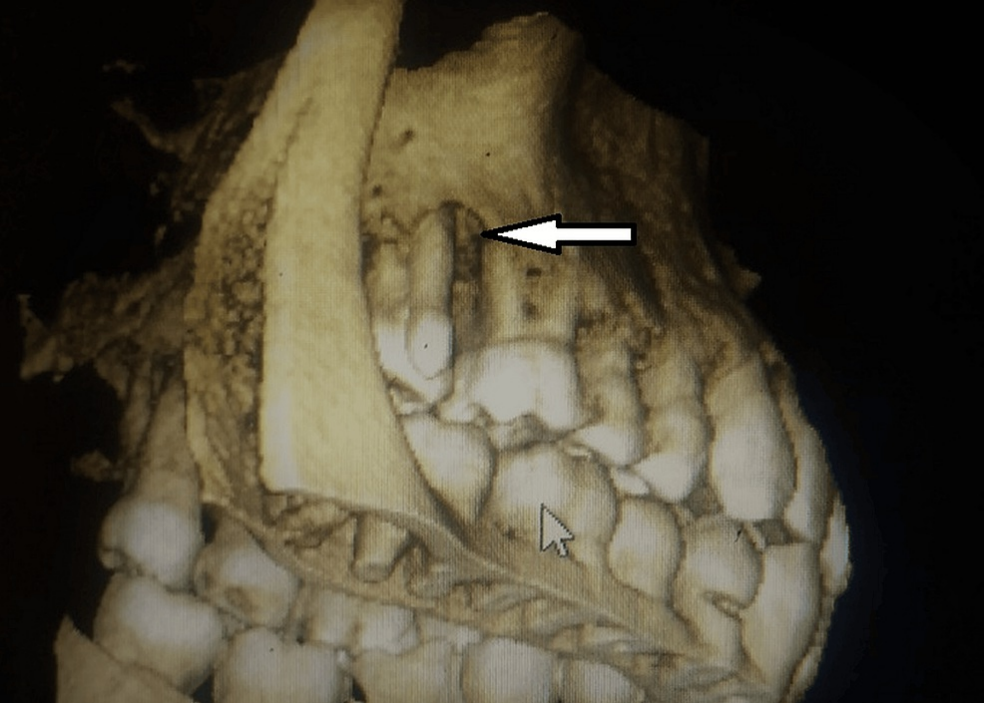
CBCT showing the periodontal bone defect CBCT: Cone beam computed tomography

A 24-year-old male reported to the department with the chief complaint of pain in the upper right back tooth region for eight months with no contributory medical history. An extra-oral examination revealed no abnormality. Intra-oral examination revealed a unilateral protostylid on the buccal aspect concerning 17, raising suspicion of fusion (due to the presence of an extra root) as a potential differential diagnosis (Figures 1-3).

The probing revealed a pocket depth of 12mm with the UNC 15 probe. A groove formation was observed on the mesial aspect of the attachment of the supernumerary cusp to the maxillary molar. Cone beam computed tomography (CBCT) was planned. Fusion is a developmental anomaly where two separate tooth buds fuse together during tooth formation, forming a single large tooth. In this case, fusion likely occurred between the maxillary molar (tooth number 17) and another adjacent tooth, resulting in the presence of an extra root and a supernumerary cusp on the buccal aspect of the molar. This condition can lead to various dental anomalies and complications, including irregular tooth morphology, abnormal root canal anatomy, periodontal defects, and increased susceptibility to dental caries and periodontal disease. The presence of an additional cusp suggests the presence of supernumerary dental structures, further complicating the tooth's morphology and function. The CBCT image revealing a periodontal bone defect indicates potential periodontal involvement associated with the fused tooth, likely due to its irregular morphology.

Additionally, the groove formation observed on the mesial aspect of the attachment of the supernumerary cusp to the maxillary molar suggests possible abnormalities in root canal anatomy, which may complicate endodontic treatment if necessary. Overall, this condition encompasses a fusion of teeth in the presence of supernumerary structures, periodontal involvement, and potential abnormalities in root canal anatomy, necessitating a multidisciplinary treatment approach involving dental, periodontal, and endodontic considerations. To understand the periodontal defect and the anatomy of the root canal. The patient was advised to undergo scaling and polishing and use a 0.2% chlorhexidine mouth rinse to limit plaque formation, followed by periodontal therapy.

## Discussion

The etiology of the protostylid is still unclear, but it is said to be due to the hyperactivity of the dental lamina, or it is believed that the PAX and MSX genes are responsible for the abnormal shape of the teeth [2]. The form of the cusp depends on the enamel thickness and its relationship to dentin. Cusp configurations depend upon the molecular patterns that are genetically determined and, on the other hand, the trial's relationship with other morphological features [3-6]. Turner and Harris suggest that such cusps arise during morphogenesis, starting from an accessory enamel knot developed at the surface where the future apex forms [7]. The finding of an extra root may have significant implications for treatment. In particular, the necessity to be prepared for a potentially complex root canal system during endodontic treatment and a less favorable periodontal prognosis due to additional roots in the fused tooth should be considered. Thus, considering the findings in this case, an extra root reportedly free-floated with no bony or tooth tissue connection correlates with the diagnosis of tooth fusion. Tooth fusion is an abnormal development in which two separate tooth buds fuse to create a single tooth with one or more extra roots.

In the present case, CBCT revealed severe bone loss and a groove formed at the junction of the cusp and molar surfaces. Various occlusal sections showed the pulp chamber and root canal anatomy, and the pulp chamber of the protostylid was closely associated with the pulp chamber of the molar. The groove formation is an area for plaque retention, leading to periodontitis. The treatment that can be advocated in such a case would be root canal therapy followed by coronoplasty and periodontal flap surgery.

Other associated problems include pit and feces caries, sensitivity, and devitalization due to the fracture. It can also interfere with occlusion and cause premature contact. It may also lead to irritation of the buccal mucosa [8]. The protostylid is a useful tool in forensic odontology since dental tissue remains unchanged even after a long stay in extreme environments [9,10]. The nomenclature used to date is mentioned (Table 1).

**Table 1:** Nomenclature used till date

Author	Terminology	Description
Bolk et al. [11]	Paramolar tubercule/Bolk’s cusp	An additional cusp on the buccal surface of molar
De Jonge-Cohen et al. [12]	Mesio-buccal edge prominences	An additional cusp on the buccal surface of molar
Dahlberg et al. [13]	Paramolar tubercule	Any stylar anomalous cusp, supernumerary inclusion on either maxillary or mandibular premolar or molars
Broom et al. [14]	Protostylid	Rudimentary external cingulum
Hlusko et al. [15]	Protostylid or Protoconidal cingulum	Interchangeable terms

Hanihara [16] gave a classification system consisting of seven grades. Based on this grading system, the protostylid in the present case was classified as grade 6. Its interference during the orthodontic banding procedures and cementation of brackets may complicate orthodontic procedures and the alignment of wires. Hence, orthodontists prefer to perform coronoplasty (selective grinding of tooth structure). Periodontal prospective accumulation of plaque leads to gingivitis, followed by periodontitis [17]. If the cusp is sharp and bulky, it may lead to irritation, keratosis, or ulceration of the buccal mucosa. Fixed prosthesis (3-unit) tooth preparation without endodontic therapy in such an abutment tooth may be difficult.

## Conclusions

Examination and other investigations may reveal variations in root canal anatomy and tooth anatomy, which play a vital role during various endodontic, periodontic, prosthodontic, and orthodontic procedures and their further course if left untreated. Furthermore, it is relevant in forensics for identification.
